# Reconstruction of Population-Level Migration Trajectories of Black-Faced Spoonbill (*Platalea minor*) Based on Citizen Science Data

**DOI:** 10.3390/ani14111663

**Published:** 2024-06-01

**Authors:** Ruilin Wang, Chang Liu, Keming Ma

**Affiliations:** 1 State Key Laboratory of Urban and Regional Ecology, Research Center for Eco-Environmental Sciences, Chinese Academy of Sciences, Beijing 100085, China; wangruilin18@mails.ucas.ac.cn (R.W.); changliu2022_st@rcees.ac.cn (C.L.); 2 College of Resources and Environment, University of Chinese Academy of Sciences, Beijing 100049, China

**Keywords:** citizen science data, LoMcT, multi-trajectory reconstruction

## Abstract

**Simple Summary:**

Understanding avian migratory routes is crucial for identifying important stopover sites during migration and for implementing targeted conservation efforts at these key locations. The Black-faced Spoonbill (*Platalea minor*) is a Class I protected species in China; however, research on it has been extremely limited to date. This study reconstructs the population-level migratory trajectories of the Black-faced Spoonbill based on citizen science data. The results indicate that Wenzhou, Xiamen, Shantou, Shanwei, Hsinchu, Chiayi, and Tainan are significant stopovers for this species. There are multiple migratory pathways for the Black-faced Spoonbill across the southeastern coastal region of China. This study also validates the application of citizen science data in restoring species migration trajectories. The findings enhance our understanding of the migratory patterns of the Black-faced Spoonbill and provide valuable insights for its conservation.

**Abstract:**

Migration is a critical ecological process for birds. Understanding avian migratory routes is essential for identifying important stopover sites and key foraging areas to ensure high-quality stopovers for birds. The Black-faced Spoonbill (*Platalea minor*), a national Grade I protected wild animal in China, is classified as endangered on the IUCN Red List of Threatened Species. Studying the migratory routes of the Black-faced Spoonbill and identifying critical stopover sites across different life histories is vital for its conservation. However, research on the migratory routes of this species has been very limited. This study, utilizing citizen science data and the Level-order-Minimum-cost-Traversal (LoMcT) algorithm, reconstructs the migratory trajectories of the Black-faced Spoonbill from 2018 to 2022. The results show that Wenzhou, Xiamen, Shantou, Shanwei, Hsinchu, Chiayi, and Tainan are significant stopovers for this species. The Black-faced Spoonbill is actively migratory during the migration season across the southeastern coastal region of China. The simulation results of this study reveal the migratory routes and activity patterns of the Black-faced Spoonbill, providing critical support for its conservation.

## 1. Introduction

Avian migration is driven by a multitude of environmental factors, resulting in the regular seasonal movements of birds between breeding and wintering grounds [1,2,3]. The migration trajectories of birds can reveal important stopover sites and critical nodes during their migration process or can elucidate the transmission pathways of highly pathogenic avian influenza [4,5]. Investigating avian migratory trajectories enables the effective determination of the timing, routes, and key stopover points of their migrations. This targeted approach provides sufficient resource support and species protection during the migration process and reduces the risk of avian influenza pandemics.

Common methods for studying avian migration include the direct observation of migratory birds, radar tracking, banding and recapture, museum specimen distribution studies, isotopic labeling, geolocator deployment and retrieval, satellite telemetry, laboratory experiments, and mathematical modeling estimates [6,7,8,9]. However, these methods sometimes offer poor resolution, often require substantial technical and financial resources, and do not provide sufficient information at the population level [10,11,12]. Historically, the study of animal migration routes has primarily relied on tracking data. Some scientists develop various computational models, algorithms, and simulations to understand migration patterns, dynamics, and the relationships between migration and environmental factors based on tracking data [13,14,15], while others conduct in-depth, population-scale studies on specific species’ migration routes, timings, and habitat preferences using extensive tracking databases [16,17]. With the exponential growth in global citizen science data on bird observations, which has greatly improved temporal and spatial resolution, scientists have made it feasible to reconstruct avian migration patterns using citizen science data.

Some studies suggest that avian migration behavior is a response to extreme climate conditions, and their migratory lifestyle may be crucial for adapting to climate change and preventing population decline [18]. Consequently, researchers initially used citizen science data combined with species distribution models to predict species distribution ranges and future distribution patterns under climate change scenarios [19,20]. Subsequently, some researchers integrated citizen science data with mechanical models, meteorological data, and land use data to reconstruct and restore the dynamics of migratory bird populations in North America during the migration season [3]. Others have employed mathematical models to calculate annual migratory trajectories, migration speeds, and changes in timing and locations for species, based on extensive bird observation records in North America. These models are enhanced by overlaying geographic coverage information and adding resistance surfaces, facilitating the exploration of avian seasonal migration strategies using citizen science data [21,22]. 

In the absence of tracking data, these methods gradually expanded the data sources for studying migration trajectories and improved the accuracy of reconstructed routes. However, a common limitation of these algorithms is their tendency to overemphasize shifts in the centroid of data distribution during population migration, leading to highly biased prediction results and failing to provide detailed migratory trajectories. This changed when researchers developed the Level-order-Minimum-cost-Traversal (LoMcT) algorithm based on the eBird dataset. This method, by clustering, grouping, and reconstructing multiple trajectories at the population level, enabled the simulation of multiple trajectories in the avian migration process [23]. This algorithm, relying solely on citizen science data, overcomes the traditional bias towards hotspots of citizen science activities, yielding scientific and reliable simulations of avian migration trajectories.

In 2021, the Black-faced Spoonbill was listed as a Class I protected species in China under the “National Key Protected Wildlife Catalogue”, and is classified as endangered by the International Union for Conservation of Nature (IUCN) Red List of Threatened Species [24,25]. Although it was common along the eastern coast of China in the 1930s, the distribution and the population size of this species have significantly declined due to water pollution and wetland destruction caused by human activities. By the 1980s, the population had decreased to fewer than 300 individuals [26]. It is the only endangered species among the six species of the family *Threskiornithidae* with a highly restricted distribution. It is generally believed to breed on the Korean Peninsula and winter in Fujian, Guangdong, Hong Kong, Macau, Taiwan, and Hainan in China, with occasional sightings in Liaoning, Shanghai, and Jiangsu, as well as in Southeast Asia including Vietnam, Thailand, and the Philippines [27,28]. Because Black-faced Spoonbills have rarely been observed historically, they are the least studied species within the *Threskiornithidae* family. Initial research on the Black-faced Spoonbill commenced only after its breeding sites were first discovered in China in 1999. Although recent years have seen systematic observations and studies on the population, distribution, and habitat of the Black-faced Spoonbill, overall, the related research remains extremely limited, making further studies both urgent and necessary [28,29].

This study employs the Level-order-Minimum-cost-Traversal (LoMcT) algorithm, using citizen science data, to simulate the population-level migratory trajectories of the Black-faced Spoonbill across common habitats in China over a five-year period. The research aims to address the following issues: (1) To what extent can the algorithm accurately reconstruct the migratory trajectories of the Black-faced Spoonbill? (2) To capture the migratory dynamics of the Black-faced Spoonbill populations within the studied regions. (3) To identify the main activity areas of the Black-faced Spoonbill. By addressing these questions, this study will provide insights into the distribution and population dynamics of the Black-faced Spoonbill and offer targeted conservation recommendations for the species.

## 2. Materials and Methods

### 2.1. Data Collection

The occurrence data for the Black-faced Spoonbill were obtained from the Global Biodiversity Information Facility (GBIF) at https://www.gbif.org/ (accessed on 10 February 2024). On the occurrence data download page, the scientific name “*Platalea minor* Temminck & Schlegel, 1849” was entered and after reviewing the annual data volume, the time range was restricted to the years 2018 to 2022, yielding a total of 36,098 records (Table 1).

### 2.2. Data Processing and Computation

The LoMcT algorithm used in this study was developed by Shi Feng et al. in 2021, comprising the following four main steps: data processing, clustering, grouping, and multi-trajectory fitting [23]. For the formulas and specific procedures, please refer to Feng’s paper [23]. In this study, Python was used for data cleaning, and Pandas was employed for data mining and trajectory reconstruction [30].

**Figure 1:**
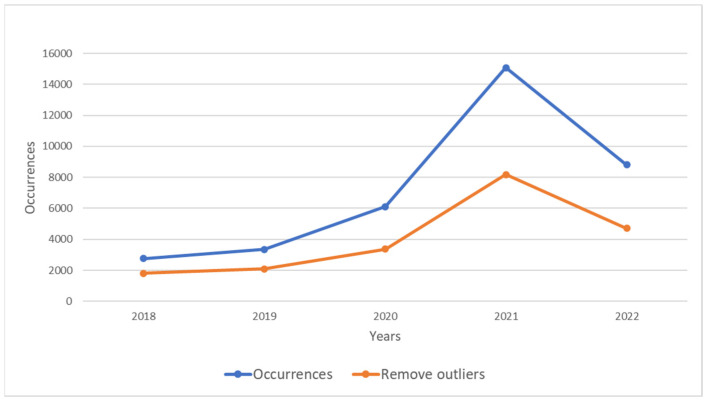
Comparison chart of raw data (Occurrences) and cleaned data (Remove outliers).

Data were cleansed and processed to retain only the species name, longitude, latitude, and observation date. To avoid redundancy during computation, duplicate records from the same day were removed. Since occurrence data based on visual observations by citizen scientists can have large positional errors, especially those falling into the sea, these were considered unreliable. Therefore, using the map coastline as a reference, points falling into the sea were removed, retaining only those on land. After initial processing, a total of 20,156 records were retained (Figure 1).

To address the absence of data on certain dates, nearest neighbor interpolation was used; for example, linear interpolation using the time and location data of the k-nearest neighbors was used to complete continuous location information. After testing, k was set to 6 in this study. To correct local biases in observation data, the Space Local Deviation Factor (SLDF) algorithm was used for anomaly detection, retaining 80% of the data sorted by SLDF values [31]. The Mean-Shift Clustering algorithm, a density-based unsupervised clustering method that ensures the stability of clusters, was used to identify the main clusters [32]. The core part of the algorithm calculated centroid distances between adjacent days, assuming that the shortest distance between two centroids had the minimum migration cost, and flight paths were determined based on the distances between adjacent centroids. Finally, a generalized additive model was used to fit longitude, latitude, and dates in chronological order, matching latitude and longitude coordinates on the same date. By connecting these coordinate points, the final migration trajectory results were formed [23].

## 3. Results

### 3.1. Migration Trajectories across Different Years

**Figure 2:**
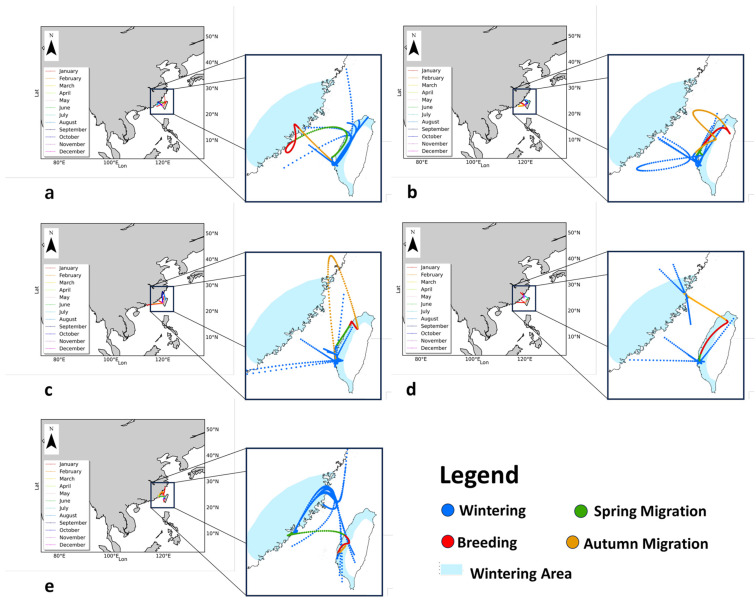
A diagram of the whole migration cycle of the Black-faced Spoonbill in 2018 to 2022. Blue lines represent the wintering migration trajectories. Red lines represent the breeding migration trajectories. Green lines represent the spring migration trajectories. Yellow lines represent the autumn migration trajectories. The map (**a**) shows the migration trajectories of the Black-faced Spoonbill in 2018; the map (**b**) shows the migration trajectories of the Black-faced Spoonbill in 2019; the map (**c**) shows the migration trajectories of the Black-faced Spoonbill in 2020; the map (**d**) shows the migration trajectories of the Black-faced Spoonbill in 2021; the map (**e**) shows the migration trajectories of the Black-faced Spoonbill in 2022.

The migratory trajectories of the Black-faced Spoonbill from 2018 to 2022 were reconstructed, showing distinct grouping patterns during the early phases of each annual migration cycle. According to the logic of the LoMcT algorithm [23], multiple migration trajectories were identified for each year. Different life histories of the species’ migration are marked with various colors (Figure 2), with reconstructed trajectories indicating that migrations in spring and autumn primarily occur over straits, while breeding season trajectories are shorter and wintering trajectories are longer and predominantly over land. The migration trajectories suggest that the Black-faced Spoonbill’s movements within the region are mostly curvilinear, likely optimizing for suitable environmental conditions, like wind speed and direction, to minimize energy expenditure. Multiple trajectory maps illustrate the dispersion or concentration within the species population during different migration periods. Convergence points on multiple trajectories likely represent ecologically valuable stopovers with abundant food supplies.

Over five years, the migration trajectories revealed that Wenzhou, Xiamen, Shantou, Shanwei, Hsinchu, Chiayi, and Tainan are critical stopovers for the Black-faced Spoonbill. The triangular area formed by Zhangzhou, Chaozhou, and Shantou, and the belt area formed by New Taipei, Taoyuan, and Hsinchu, serve as breeding areas, while the belt formed by Xiamen, Quanzhou, Putian, and Fuzhou, and the western coastline of Taiwan, serve as wintering areas. Overall, the activity range during the breeding season is the smallest, while the range during the wintering and autumn migration periods is the largest.

**Figure 3:**
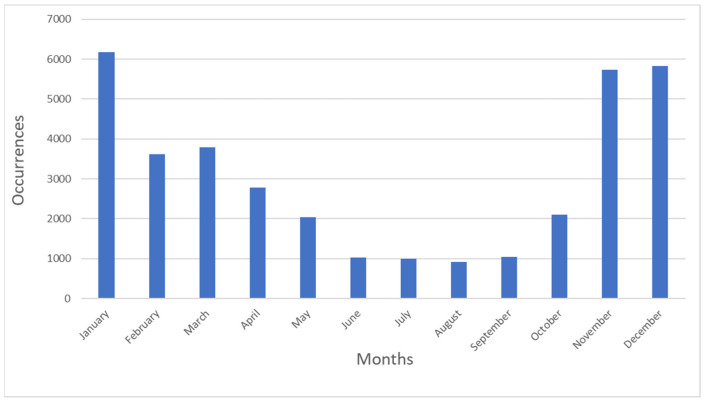
Monthly occurrence records of the Black-faced Spoonbill over five years.

According to current knowledge, most of the Black-faced Spoonbills residing in China’s southeastern coastal area are residents [33]. However, the data, showing certain fluidity in monthly occurrence records (Figure 3), indicate that a portion of the population within China is migratory. Our migration trajectory reconstructions also reflect that during certain periods each year, multiple populations form distinct migration trajectories.

### 3.2. Analysis of Departure Times and Flight Speeds

**Figure 4:**
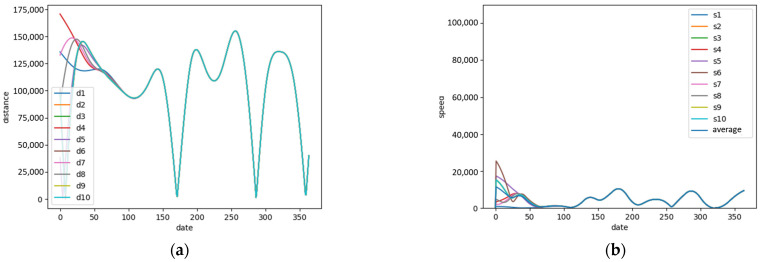
(**a**) Offset distances graph of Black-faced Spoonbills in 2018. d1 represents group1, d2 represents group2, and so forth. (**b**) Speed graph of Black-faced Spoonbill in 2018. s1 represents group1, s2 represents group2, and so forth.

We calculated the deviation distances and migration speeds of the Black-faced Spoonbill during each migration. For instance, in 2018, Figure 4a shows the daily Euclidean deviation distances during the migration process. The x-axis represents the day of the year, while the y-axis shows the periodic changes in the deviation distance between the trajectory positions and a straight line connecting the start and end points on each date in the Mercator coordinate system. Figure 4b shows the population migration speed of the Black-faced Spoonbill in 2018. From these graphs, it is observed that at around day 170, approximately in late June, the Black-faced Spoonbills reach their breeding grounds; by day 270, around late September, they leave the breeding area to commence autumn migration. They arrive at the wintering grounds around day 320, approximately mid-November (see Appendix A for charts of other years). A summary table of migration start and end times across other years is provided (Table 2).

## 4. Discussion

This study represents the first use of citizen science data to simulate the population-level migratory trajectories of the Black-faced Spoonbill, demonstrating that populations of this species undergo seasonal migrations throughout the year in China’s southeastern coastal region. At the population level, this species exhibits multiple migratory routes; typically beginning spring migration from February to April, reaching breeding sites by June; and starting autumn migration in either July or September, with most arriving at wintering grounds by November.

The Black-faced Spoonbill is an endangered species and research on it started late, with the least amount of studies among Threskiornithidae birds [28,34]. Some sources suggest that in Fujian, China, the Black-faced Spoonbill is a resident bird, though it is extremely rare [28]. Although the trajectory simulations of this study have shown multiple migratory paths, they are mainly concentrated in the southeastern coastal region of China, with no results for broader areas. This phenomenon may be due to the fact that although species distribution records indicate the presence of Black-faced Spoonbills in Russia, Korea, China, and Southeast Asia [35], they are rarely found in these locations in reality. Without tracking data, understanding the complete migratory routes through the regions of Russia, Korea, China, and Southeast Asia presents practical challenges. While the methods used in this paper have been validated as credible, the inherent limitations of citizen science data mean that reconstructing species migration trajectories based solely on this data inevitably faces constraints.

Based on the deviation distances and migration speeds during the Black-faced Spoonbill’s migration, we inferred the annual start and end times of the migratory behavior for the population. However, as seen from the results in Table 2, the start and end times for 2022 differ significantly from other years. To find the cause, we examined the temperature data for the southeastern coastal region of China for the corresponding year and discovered that the temperatures in 2022 were lower than in previous years, which could be the reason for the different migration timing of the Black-faced Spoonbill population. This finding also demonstrates that the results obtained from citizen science data can accurately reflect the annual temperature changes, indirectly validating the reliability of our findings.

It is commonly believed that species tend to follow the shortest path during migration; if a migratory path is curved, it suggests that the species has chosen to detour to avoid obstacles [36]. Based on this principle, by examining our trajectory lines, it can be determined that areas where multiple migration trajectories are concentrated are important stopover sites during the migration. Therefore, we identify Wenzhou, Xiamen, Shantou, Shanwei, Hsinchu, Chiayi, and Tainan as critical stopovers for the Black-faced Spoonbill. The triangular area formed by Zhangzhou, Chaozhou, and Shantou, and the belt-like region formed by New Taipei, Taoyuan, and Hsinchu, serve as breeding areas, while the belt-like region formed by Xiamen, Quanzhou, Putian, and Fuzhou, along with the western coast of Taiwan, serve as wintering areas. Monthly frequency data show that the Black-faced Spoonbill is more frequently observed during the wintering season and is observed less so during the breeding season. This may lead to our reconstructed trajectories showing a smaller range of activity during the breeding period and larger ranges during the wintering and autumn migration periods.

Overall, our study demonstrates that citizen science data can be applied to the simulation of species population-level migration trajectories, challenging the long-standing reliance on tracking data for studying species migration. As citizen science data continue to accumulate, this method has the potential to be applied to more species, helping to fill gaps in the human understanding of migration trajectories for many species. However, we must acknowledge that due to the limitations of citizen science data, our results are not as accurate as those obtained from actual migration tracking. Yet, tracking data only reflect individual trajectories and cannot represent the whole population. Therefore, future research should combine avian tracking data with citizen science data to optimally reconstruct avian migration trajectories.

## 5. Conclusions

This study utilized the LoMcT algorithm and citizen science data to simulate the migratory routes of the Black-faced Spoonbill. The results show that this algorithm can simulate multiple migratory trajectories at the population level for the Black-faced Spoonbill. In the absence of tracking data, this method currently offers an advanced means of capturing the migratory dynamics of the Black-faced Spoonbill population. The triangular area formed by Zhangzhou, Chaozhou, and Shantou, along with the belt-like region formed by New Taipei, Taoyuan, and Hsinchu, serve as breeding areas, while Xiamen, Quanzhou, Putian, and Fuzhou, along with the western coast of Taiwan, serve as wintering areas. The results of this study are largely consistent with those obtained by JIA et al. based on tracking data [26], indicating that the Black-faced Spoonbill primarily migrates along the eastern coastal regions of China. Due to the limited availability of tracking data, other studies have either failed to provide detailed migration routes or have only reconstructed inland migration paths. In contrast, this study utilizes extensive citizen science data to reconstruct the migration trajectories within the active regions of the Black-faced Spoonbill. In future design and management plans for nature reserves for endangered waterbirds such as the Black-faced Spoonbill, it is essential to consider the combined effects of seasonal changes and landscape connectivity. Integrating new technologies such as satellite remote sensing, camera traps, and geographic information systems (GISs) will be crucial. A multidisciplinary approach should be adopted to conduct targeted conservation and planning.

## Figures and Tables

**Table 1:** Data of Black-faced Spoonbill from 2018 to 2022 in GBIF.

Occurrences Per Dataset	Count
EOD—eBird Observation Dataset	22,354
2021_nsmk_biodiversity_observations_bird_197960	5784
2021_nsmk_biodiversity_observations_bird_136784	5784
Bird survey of Budai salt pans in Taiwan	812
iNaturalist Research-grade Observations	787
Taiwan New Year Bird Count	298
Ecological Records of Birds in Chenglong Wetlands	86
Dataset of long-term bird monitoring in Qigu wetlands	59
Observation.org, Nature data from around the World	53
Occurrence dataset of waterbirds in Tiaozini Wetland	39
Biological Collection of the National Taiwan Museum	1
The Survey Data of Mianhua and Huaping Islets Wildlife Refuge	1
Occurrence dataset of birds in the Sihong Hongze Lake Wetland National Nature Reserve	1

**Table 2:** Annual start and end month of the migratory behavior for the Black-faced Spoonbill population.

Year	Spring Migration		Autumn Migration	
	Start Month	End Month	Start Month	Start Month
2018	March	June	September	November
2019	March	May	September	November
2020	March	June	September	November
2021	April	June	July	November
2022	February	May	July	September

**Figure A1:**
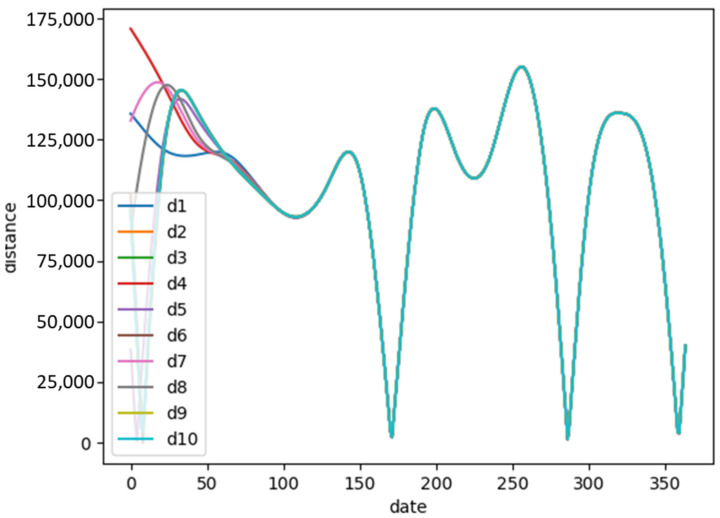
Offset distances graph of Black-faced Spoonbill in 2018.

**Figure A2:**
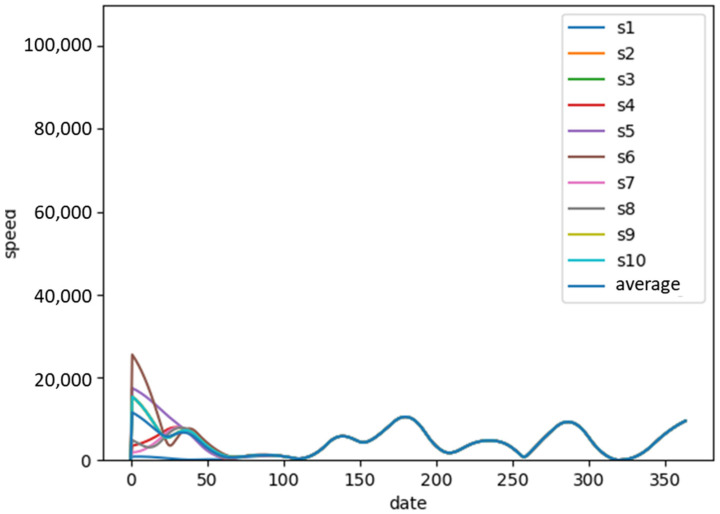
Speed graph of Black-faced Spoonbills in 2018.

**Figure A3:**
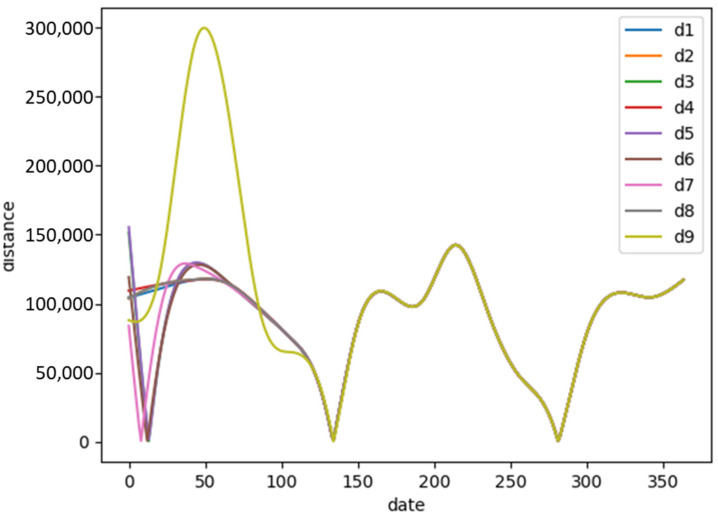
Offset distances graph of Black-faced Spoonbills in 2019.

**Figure A4:**
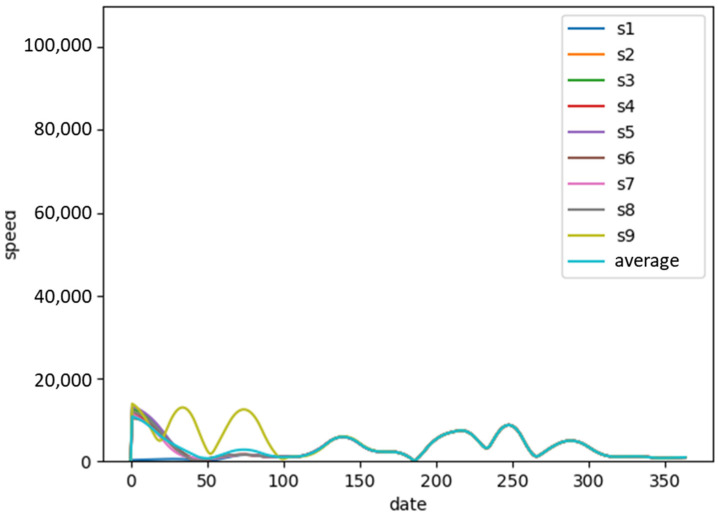
Speed graph of Black-faced Spoonbills in 2019.

**Figure A5:**
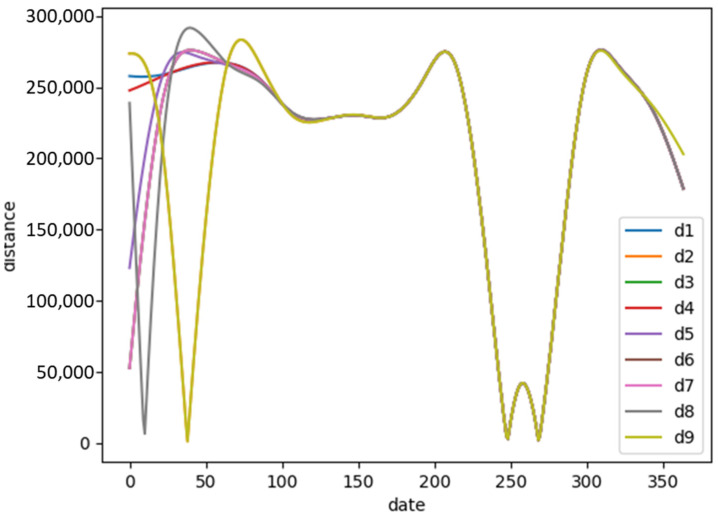
Offset distances graph of Black-faced Spoonbills in 2020.

**Figure A6:**
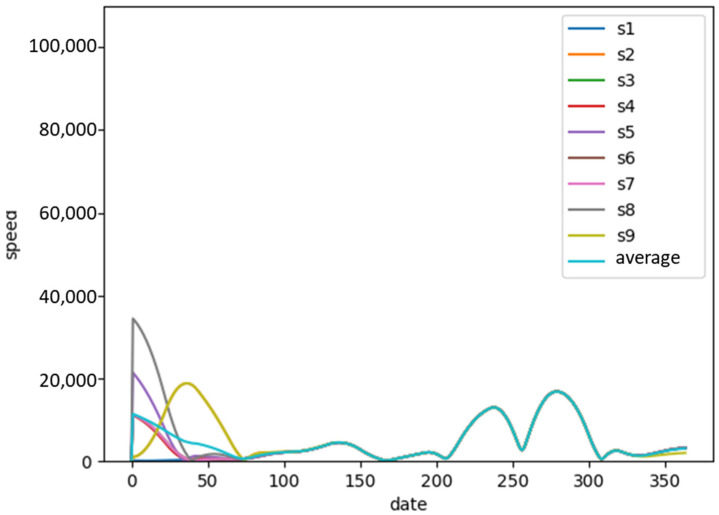
Speed graph of Black-faced Spoonbills in 2020.

**Figure A7:**
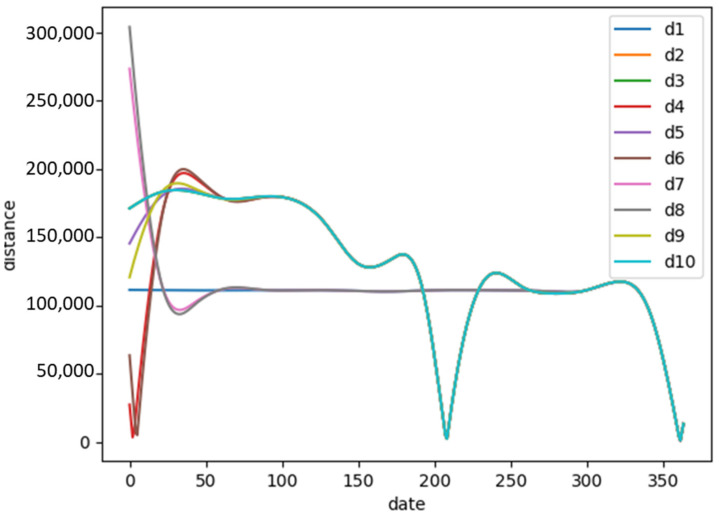
Offset distances graph of Black-faced Spoonbills in 2021.

**Figure A8:**
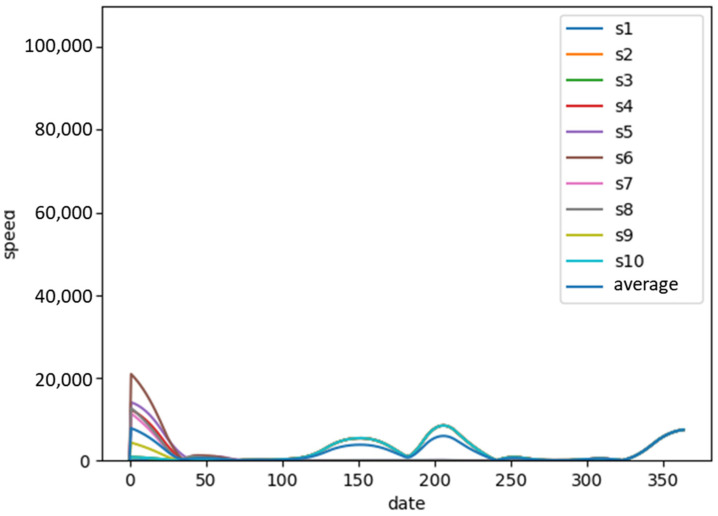
Speed graph of Black-faced Spoonbills in 2021.

**Figure A9:**
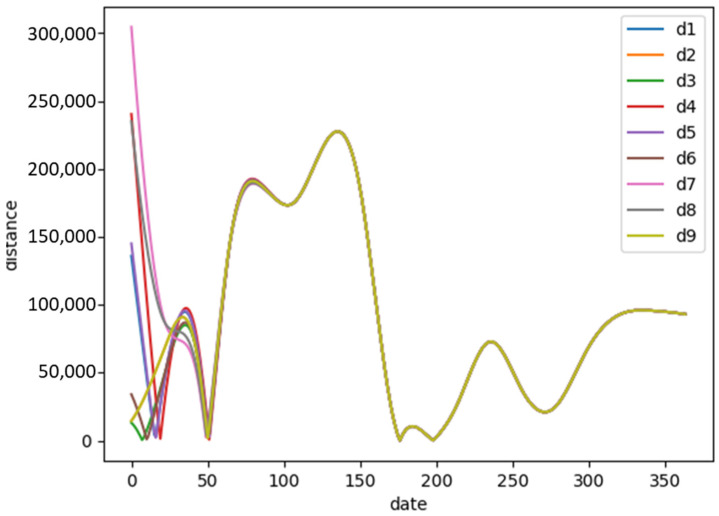
Offset distances graph of Black-faced Spoonbills in 2022.

**Figure A10:**
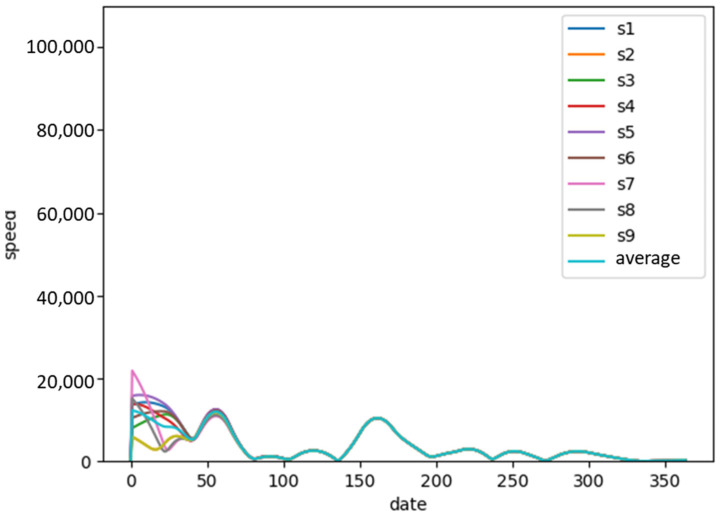
Speed graph of Black-faced Spoonbills in 2022.

**Figure A11:**
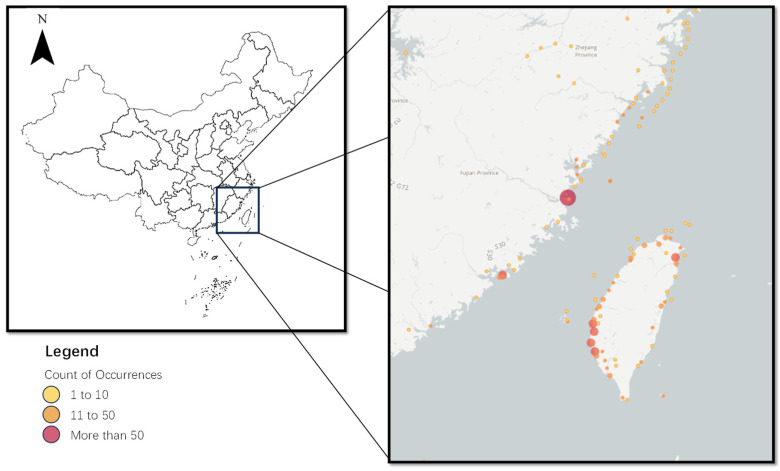
Schematic diagram of the species occurrence data distribution. Points that fall on the ocean are considered to have inaccurate coordinates and will be excluded. Only points located on land will be retained.

## Data Availability

Data used in this study are available through a search on GBIF (https://www.gbif.org/) (access on 20 February 2024).
